# 单倍体造血干细胞移植治疗合并吉尔伯特综合征血液系统恶性肿瘤9例临床分析

**DOI:** 10.3760/cma.j.cn121090-20240311-00088

**Published:** 2024-09

**Authors:** 晓璐 朱, 景枝 王, 萌 吕, 婷婷 韩, 凤美 郑, 育红 陈, 圆圆 张, 欢 陈, 晓辉 张, 兰平 许, 晓军 黄, 昱 王

**Affiliations:** 北京大学人民医院，北京大学血液病研究所，国家血液系统疾病临床医学研究中心，造血干细胞移植治疗血液病北京市重点实验室，北京 100044 Peking University People's Hospital, Peking University Institute of Hematology, National Clinical Research Center for Hematologic Disease, Beijing Key Laboratory of Hematopoietic Stem Cell Transplantation, Beijing 100044, China

## Abstract

2013年1月1日至2024年3月1日，9例合并吉尔伯特综合征的血液恶性肿瘤患者在北京大学血液病研究所接受单倍体造血干细胞移植。男7例，女2例，中位年龄38（13～60）岁。急性髓系白血病3例，急性淋巴细胞白血病3例，骨髓增生异常综合征2例，慢性粒-单核细胞白血病1例。所有患者均未合并病毒性肝炎。9例患者中，7例采用白消安+环磷酰胺+抗胸腺细胞球蛋白预处理方案，2例采用全身放射治疗+环磷酰胺+抗胸腺细胞球蛋白预处理方案。所有患者均获得粒细胞植入，8例患者获得血小板植入。所有患者移植前中位血清总胆红素为45.4（22.5～71.2）µmol/L，预处理前1 d为22.0（18.0～37.2）µmol/L。8例患者在移植后20 d血清总胆红素低于基线值，1例患者移植后5 d总胆红素水平一过性升高，考虑白消安预处理毒性所致。所有患者均未并发肝小静脉闭塞病。中位随访时间739（42～2 491）d。随访期内，复发死亡1例，余8例患者均无病生存。

## Abstract (en)

From January 1, 2013, to March 1, 2024, nine patients with hematological malignancies complicated by Gilbert's syndrome in Peking University People's Hospital underwent allogeneic hematopoietic stem cell transplantation（allo-HSCT）. The patients comprised seven male and two female cases, with a median age of 38（13–60）years old. Among them, three cases were acute myeloid leukemia, three cases were acute lymphocytic leukemia, two cases were myelodysplastic syndrome, and one case was chronic myelomonocytic leukemia. None of the patients had viral hepatitis. Of the nine cases, seven cases received the Bu-Cy+ATG regimen, while the other two cases received the TBI-Cy+ATG regimen（Bu, busulfan; Cy, cyclophosphamide; ATG, antithymocyte immunoglobulin; and TBI, total body irradiation）. All patients achieved neutrophil engraftment, and eight received platelet engraftment. The median total bilirubin level was 45.4（22.5–71.2）µmol/L before transplantation and 22.0（18.0–37.2）µmol/L on −1d of preconditioning. The total bilirubin level on +20d after the transplantation of eight patients decreased compared with the baseline level before transplantation. Moreover, one patient had a transient increase in the total bilirubin level on +5d after transplantation, which was considered to be attributed to the toxicity of Bu. No patients were complicated by hepatic veno-occlusive disease. The median follow-up time was 739（42–2 491）days. During the follow-up period, one patient died of recurrence, and the remaining eight patients had disease-free survival events.

吉尔伯特综合征（Gilbert's syndrome）又名体质性肝功能不良、遗传性非溶血性高胆红素血症，发病率为3％～10％[1]。患者通常无明显症状，一般情况良好，临床以慢性或复发性黄疸为特征。吉尔伯特综合征的分子基础在于肝脏中胆红素与葡萄糖醛酸结合的缺陷，而尿苷二磷酸葡萄糖醛酸转移酶（UGT1A1）起到了关键的催化作用。UGT1A1基因突变导致胆红素糖醛基化障碍，引起发病[2]。研究证实部分药物可以通过抑制UGT1A1基因表达或竞争性抑制UGT1A1酶来发挥治疗作用，从而导致外源性生物转化受损，引起潜在的不良反应[2]。目前绝大多数造血干细胞移植预处理采用为含白消安（Bu）方案，既往研究提示含Bu清髓预处理会增加合并吉尔伯特综合征血液恶性肿瘤患者的总死亡率和非复发死亡率[3]。合并吉尔伯特综合征的血液恶性肿瘤患者能否耐受含Bu预处理异基因造血干细胞移植（尤其是单倍体移植），国内尚未见相关报道。为了探讨单倍体造血干细胞移植治疗合并吉尔伯特综合征血液系统恶性肿瘤患者的安全性，本研究对北京大学血液病研究所接受单倍体移植的合并吉尔伯特综合征的血液恶性肿瘤病例进行回顾性分析。

## 病例与方法

一、病例

本研究纳入2013年1月1日至2024年3月1日在北京大学人民医院接受allo-HSCT的合并吉尔伯特综合征血液恶性肿瘤患者9例。以检索病历方式获取患者年龄、性别、血液原发疾病诊断、供受者性别及血型、人类白细胞抗原（HLA）配型、干细胞来源、预处理方案［是否含Bu、抗胸腺细胞球蛋白（ATG）］、移植前合并症、移植前后胆红素水平、造血细胞移植合并症指数（HCT-CI）评分、粒细胞及血小板植入时间、单个核细胞（MNC）和CD34^+^细胞输注量等临床信息。本研究经北京大学人民医院伦理委员会批准（2020PHB37601），所有患者均签署知情同意书。

二、定义

吉尔伯特综合征的诊断为排除性[2]，结合患者病史、临床表现、家族史及辅助检查（证实UGT1A1突变），排除其他导致胆红素升高的原因可考虑诊断。粒细胞植入定义为连续3 d外周血中性粒细胞绝对计数（ANC）≥0.5×10^9^/L，血小板植入定义为连续7 d血小板计数≥20×10^9^/L且脱离血小板输注。HCTCI参照相应标准计算[4]。急性移植物抗宿主病（aGVHD）分度及分级参照改良Glucksberg分度法[5]。肝小静脉闭塞病（VOD）诊断参照主要依据2016年欧洲骨髓移植学会（EBMT）标准[6]。非复发死亡是指与疾病复发/进展无关的死亡。

三、随访

采用查阅门诊/住院病历和电话随访方式获得患者移植后生存情况。随访截止时间为2024年3月1日。

## 结果

一、患者一般情况

9例患者中男7例，女2例，中位移植年龄38（13～60）岁。急性髓系白血病（AML）3例，急性淋巴细胞白血病3例，骨髓增生异常综合征2例，慢性粒-单核细胞白血病1例。所有患者均未合并病毒性肝炎。基线特征信息见表1。

**表1:** 9例接受单倍体造血干细胞移植合并吉尔伯特综合征血液系统恶性肿瘤患者的基线特征

例号	性别	年龄（岁）	诊断	UGT1A1基因突变位点	BMI（kg/m^2^）	脂肪肝	总胆红素（µmol/L）	间接胆红素（µmol/L）
1	男	32	ALL	c.211G>A、c.1456T>G	21.9	无	22.5	12.5
2	男	33	AML	c.211G>A、c.-40_-39insTA	23.8	有	55.2	38.7
3	男	40	MDS	c.211G>A	21.6	无	57.8	37.9
4	女	25	MDS	c.-3279T>G	18.4	无	71.2	59.6
5	男	46	AML	c.211G>A	22.2	无	30.8	15.8
6	男	16	MDS	c.211G>A、c.-40_-39insTA	23.6	无	45.4	26.6
7	女	43	AML	c.-3279T>G	20.3	无	40.7	31.5
8	男	38	ALL	c.-40_-39insTA、c.-3279T>G	24.8	无	54.5	36.8
9	男	56	CMML	c.211G>A	17.5	无	32.9	20.9

**注** BMI：体重指数；HLA：人类白细胞抗原；AML：急性髓系白血病；ALL：急性淋巴细胞白血病；MDS：骨髓增生异常综合征；CMML：慢性粒-单核细胞白血病

二、预处理

9例患者中7例应用Bu-环磷酰胺（Cy）+ATG方案（Bu 0.8 mg/kg每6 h 1次静脉滴注，−8 d～−6 d），2例应用全身放射（TBI）-Cy+ATG方案（表1）。供受者性别不合3例，供受者血型不合4例（其中主要不合3例，次要不合1例）。外周血干细胞移植7例，外周血干细胞移植联合骨髓移植2例。预处理期间，3例患者接受泊沙康唑一级真菌预防，6例接受卡泊芬净一级真菌预防；7例患者移植预处理接受复方磺胺甲唑预防肺孢子菌肺炎。所有患者均使用前列腺素E1预防VOD。

三、造血重建

所有患者均获得粒细胞植入，中位植入时间为移植后13（12～20）d。8例患者获得血小板植入，中位植入时间为移植后18.5（12～27）d，1例患者（例5）未获血小板植入［原发病AML，移植前疾病状态未缓解（NR），移植后2个月血液学复发］。详见表2。

**表2:** 9例合并吉尔伯特综合征血液系统恶性肿瘤患者的造血干细胞移植特征及随访结果

例号	移植前疾病状态	HCT-CI	HLA配型	预处理方案	供者性别	ABO血型（供者/受者）	MNC（×10^8^/kg）	CD34^+^细胞（×10^6^/kg）	粒细胞植入（d）	血小板植入（d）	移植前苯巴比妥	真菌预防	SMZ预防	移植后苯巴比妥	保肝药物	生存时间（d）
1	CR1	0	5/10	Bu-Cy+ATG	男	B/B	7.22	4.32	+13	+18	否	泊沙康唑	是	否	无	174
2	CR1	0	5/10	Bu-Cy+ATG	男	A/A	11.38	2.00	+13	+19	否	泊沙康唑	是	否	异甘草酸镁	313
3	/	0	5/10	Bu-Cy+ATG	男	O/O	9.56	1.69	+20	+27	否	卡泊芬净	是	否	多烯磷脂酰胆碱	1 930
4	/	0	5/10	TBI-Cy+ATG	女	O/O	13.03	4.93	+14	+12	否	卡泊芬净	是	否	异甘草酸镁	1 166
5	NR	1	5/10	TBI-Cy+ATG	女	B/O	6.62	3.05	+18	未植活	否	卡泊芬净	否	否	异甘草酸镁	125^a^
6	/	0	5/10	Bu-Cy+ATG	男	B/B	10.93	6.03	+12	+13	是	泊沙康唑	否	是	无	80
7	CR1	0	5/10	Bu-Cy+ATG	男	A/AB	7.11	8.63	+13	+13	是	卡泊芬净	是	是	异甘草酸镁	42
8	CR1	2	5/10	Bu-Cy+ATG	男	B/O	5.56	2.74	+13	+20	否	卡泊芬净	否	否	无	1 769
9	/	5	5/10	Bu-Cy+ATG	女	B/O	9.10	2.86	+20	+21	否	卡泊芬净	是	否	复方甘草酸苷	2 491

**注** HCT-CI：造血干细胞移植合并症指数；CR1：第1次完全缓解；NR：未缓解；SMZ：复方磺胺甲唑；MNC：单个核细胞；ATG：抗胸腺细胞球蛋白；Bu：白消安；Cy：环磷酰胺；TBI：全身放射治疗；/：不适用；^a^例5移植后2个月血液学复发，3.5个月死亡

四、移植前后胆红素水平

所有患者在预处理期间总胆红素水平均较移植前基线水平下降。移植前中位总胆红素水平为45.4（22.5～71.2）µmol/L，预处理前1 d为22.0（18.0～37.2）µmol/L，其中2例在预处理期间接受了苯巴比妥治疗。

8例患者在移植后20 d内总胆红素水平较基线下降。移植后20 d中位总胆红素水平为22.0（15.1～27.4）µmol/L。1例患者（例1）移植后5 d总胆红素水平升高至移植前基线水平的2倍，在移植后13 d胆红素水平降至基线值以下。该患者应用含Bu预处理方案，预处理期间应用复方磺胺甲唑及泊沙康唑，未使用苯巴比妥，胆红素升高考虑预处理毒性所致。所有患者均未并发VOD。

7例患者随访至移植后100 d。4例移植后总胆红素水平均未超过移植前基线水平（例2、3、4、8）。1例患者（例1）在移植后45 d总胆红素水平升高至移植前基线水平的2倍，停用达沙替尼后总胆红素水平恢复至移植前基线水平。1例患者（例9）在移植后55 d总胆红素水平升高至移植前基线水平的2倍，考虑aGVHD，经抗GVHD治疗胆红素水平降至移植前基线水平。1例患者（例5）移植后2个月血液学复发，移植后90 d总胆红素水平升高至移植前基线水平的2.5倍，考虑消化道出血、治疗性供者淋巴细胞输注（DLI）后aGVHD。

5例患者随访至移植后200 d。1例患者（例1）在移植后125 d总胆红素升高至移植前基线水平的2倍，考虑达沙替尼不良反应，在停用达沙替尼后胆红素恢复至移植前基线水平。余4例患者（例2、3、4、8）移植后总胆红素均未超过移植前基线水平。

五、GVHD发生情况

本研究9例患者采用环孢素A+短程甲氨蝶呤+霉酚酸酯（MMF）方案进行GVHD预防。随访期内，除1例（例5）预防性DLI后发生GVHD，1例患者发生Ⅲ度aGVHD，2例患者发生慢性GVHD。例9于移植后55 d发生Ⅲ度aGVHD，予以甲泼尼龙1 mg·kg^−1^·d^−1^治疗7 d后获得完全缓解（CR），23 d后胆红素降至移植前基线水平。8例患者移植后90 d内环孢素A剂量70～250 mg/d，4例胆红素水平均未超过移植前基线水平，期间环孢素A血药浓度为136～313 µg/L；2例患者胆红素水平最高为移植前基线水平的2倍，期间环孢素A血药浓度为154～298 µg/L；2例患者胆红素最高为移植前基线水平的1.5倍，期间环孢素A血药浓度为148～308 µg/L。

六、随访

中位随访739（42～2 491）d。随访期内，复发死亡1例（例5，原发病AML，移植前疾病状态NR，移植后2个月血液学复发），余8例患者均无病生存。

## 讨论

吉尔伯特综合征是常染色体显性遗传性良性疾病，由于患者体内UGT1A1基因启动子中的纯合多态性A（TA）7TAA插入启动子中，使UGT活性降低，导致间接胆红素不能在肝脏完全与葡萄糖醛酸结合而转化成结合胆红素,引起血液中间接胆红素增高[2]。年龄、性别、种族、吸烟史、肥胖、酒精摄入、药物、心理压力等多种因素可影响吉尔伯特综合征患者的胆红素水平[2]。化学药物（如伊立替康）、抗病毒药物（如阿扎那韦、茚地那韦）、酪氨酸激酶抑制剂（TKI）、非甾体类抗炎药（NSAID）、环孢素A等可通过抑制或竞争UGT1A1活性从而影响胆红素代谢。

吉尔伯特综合征患者是否能耐受allo-HSCT以及预处理方案选择目前尚未达成共识[7]。McDonald等[3]回顾性分析了3 379例造血干细胞移植（1 855例allo-HSCT，1 524例auto-HSCT），其中211例患者合并吉尔伯特综合征。结果提示合并吉尔伯特综合征组应用含Bu清髓预处理方案的总死亡率较未合并组升高2.3倍，非复发死亡率升高2.8倍。而应用含TBI/美法仑预处理方案对总死亡率和非复发死亡率无显著影响。但两组的死亡原因构成无明显差异。Yu等[8]报导1例应用含Bu方案的全相合无关供者造血干细胞移植，患者耐受可，随访至移植后510 d，患者仍处于无病生存状态。本研究中，7例患者应用含Bu预处理方案，所有患者在预处理前1 d内胆红素均较移植前基线水平下降，6例患者在移植后20 d内胆红素水平均较基线水平下降，初步提示合并吉尔伯特综合征的血液肿瘤患者对Bu方案耐受良好。推测其原因可能是：①体内外研究表明间接胆红素具有抗氧化作用，对从因接受造血干细胞移植预处理而处于氧化应激状态的脏器具有保护作用[9]–[11]；②McDonald等[3]研究中Bu为口服制剂，本组病例预处理方案中Bu均为静脉给药，不存在首过效应，其毒性可能更低[12]–[13]；目前国际经典方案推荐Bu总量为12.8 mg/kg，我中心为9.6 mg/kg[14]，患者发生预处理毒性风险更小；③苯妥英钠作为UGT1A1诱导剂预防高胆红素血症的发生[15],本研究中应用含Bu预处理的患者均在预处理期间接受了苯妥英钠治疗。

研究表明预处理方案中的Bu、Cy、TBI等可能会导致VOD的发生。上述药物或方案对窦隙内皮细胞（SEC）和肝细胞的毒性损伤是VOD发生的直接原因，肝小叶中心区域（肝腺泡3区）因缺乏谷胱甘肽（GSH）而更易发生损伤[16]–[18]。allo-HSCT后VOD发生率为8.9％～14.0％[19]–[23]。鲜有研究报导合并吉尔伯特综合征的患者移植后VOD发生情况。研究者将在造血干细胞移植后20 d内直接胆红素85.5～169.3 µmol/L假定为中度VOD，将直接胆红素水平≥171 µmol/L假定为重度VOD，分析发现合并吉尔伯特综合征组重度VOD的发生率为9％（19/211），而未合并组发生率为5％（157/3 168）[3]。但这种假定不完全符合VOD的诊断标准，这也是该研究的局限性之一。本研究9例患者中7例应用含Bu预处理方案，2例应用含TBI预处理方案，且所有患者均未达到肥胖标准，仅1例合并脂肪肝，移植后均未发生VOD，另外上述讨论的个案报道中的患者亦未发生VOD[8]。目前国际上尚未形成统一的VOD预防方案，随机对照临床试验（RCT）及荟萃分析显示熊去氧胆酸可降低移植后VOD发生率[24]–[26]，而普通肝素或低分子量肝素是否具有预防作用效果不一[27]–[29]，去纤苷是目前国外唯一获批的VOD治疗药物，尚未批准用于预防，部分关于预防的RCT研究结果令人鼓舞[30]–[32]，而关于前列腺素E1的RCT研究缺少一致性结论[33]–[35]。本研究中，所有患者均采用了前列腺素E1常规预防，6例患者应用了异甘草酸镁、多烯磷脂酰胆碱、复方甘草酸苷保肝药物，保肝药物的运用与胆红素水平未观察到相关性。

在本研究中，除1例复发死亡，其余病例在控制药物、aGVHD等因素后，胆红素水平均较移植前处于稳定状态，初步提示合并吉尔伯特综合征的血液恶性肿瘤患者对单倍体移植具有良好的耐受性。但本研究病例样本量较少、部分病例随访时间较短，未来应开展前瞻性临床研究纳入更大样本量验证其安全性。
